# The Combination of Liposomes and Metallic Nanoparticles as Multifunctional Nanostructures in the Therapy and Medical Imaging—A Review

**DOI:** 10.3390/ijms22126229

**Published:** 2021-06-09

**Authors:** Marika Musielak, Jakub Potoczny, Agnieszka Boś-Liedke, Maciej Kozak

**Affiliations:** 1 Department of Electroradiology, Poznan University of Medical Sciences, 61-701 Poznań, Poland; 2 Radiobiology Laboratory, Department of Medical Physics, Greater Poland Cancer Centre, 61-866 Poznań, Poland; 3 Department of Macromolecular Physics, Faculty of Physics, Adam Mickiewicz University, 61-614 Poznań, Poland; agnieszkaboss@gmail.com (A.B.-L.); mkozak@amu.edu.pl (M.K.); 4 Heliodor Swiecicki Clinical Hospital in Poznan, 60-355 Poznań, Poland; potocznyjakub@op.pl

**Keywords:** liposomes, nanotechnology, nanotheragnostics, metallic nanoparticles

## Abstract

Nanotechnology has introduced a new quality and has definitely developed the possibilities of treating and diagnosing various diseases. One of the scientists’ interests is liposomes and metallic nanoparticles (LipoMNPs)—the combination of which has introduced new properties and applications. However, the field of creating hybrid nanostructures consisting of liposomes and metallic nanoparticles is relatively little understood. The purpose of this review was to compile the latest reports in the field of treatment and medical imaging using of LipoMNPs. The authors focused on presenting this issue in the direction of improving the used conventional treatment and imaging methods. Most of all, the nature of bio-interactions between nanostructures and cells is not sufficiently taken into account. As a result, overcoming the existing limitations in the implementation of such solutions in the clinic is difficult. We concluded that hybrid nanostructures are used in a very wide range, especially in the treatment of cancer and magnetic resonance imaging. There were also solutions that combine treatments with simultaneous imaging, creating a theragnostic approach. In the future, researchers should focus on the description of the biological interactions and the long-term effects of the nanostructures to use LipoMNPs in the treatment of patients.

## 1. Introduction

At the core of the potential applications of lipid systems, and in particular liposomes, in medicine, is the ability of amphiphilic lipid molecules to self-organize and create a wide range of 3D structures. In aqueous environments, these molecules typically can form structures of a spherical shape (liposomes or micelles), various lamellar structures, or numerous phases characterized by cubic or hexagonal symmetry [1]. The structure of these systems and the conditions of their formation in the presence of a wide range of substances were characterized in detail using a wide range of physical methods [2,3]. Since 1970, liposomes (LPs) have been extensively studied in the category of a drug delivery system (DDS) due to their high compatibility and ability to deliver hydrophilic and hydrophobic substances [4,5].

**Figure 1:**
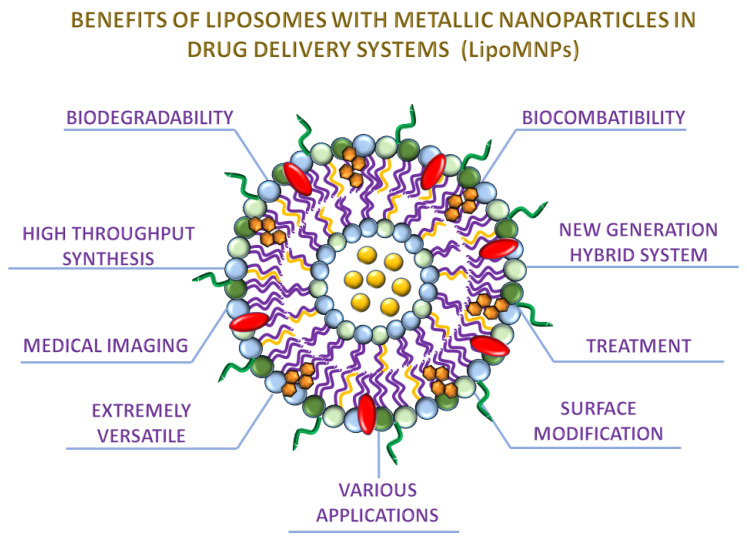
Benefits of liposomes with incorporated metallic nanoparticles in drug delivery systems. The picture presents the most interesting and crucial aspects of LipoMNPs according to usage in cancer treatment and medical imaging. The liposome of mixed lipid composition was selected as a simplified model. The Lipo-MNPs consist of phospholipids, cholesterol, DSPE-PEG, and MNPs.

Liposomes have been prepared as spherical nanostructures with an aqueous inner core surrounded by bilayers of phospholipids [6,7]. Moreover, LPs are at the center of interest related to nanotechnology because of the variety of multiple kinds of implementations [8,9] (Figure 1).

An important application area of liposomes that have dominated their potential applications for a long time has been drug or genetic material transfer systems. Currently, the interest in such applications, especially as carriers of vaccines or therapeutic nucleic acids, is still serious [10,11,12].

**Figure 2:**
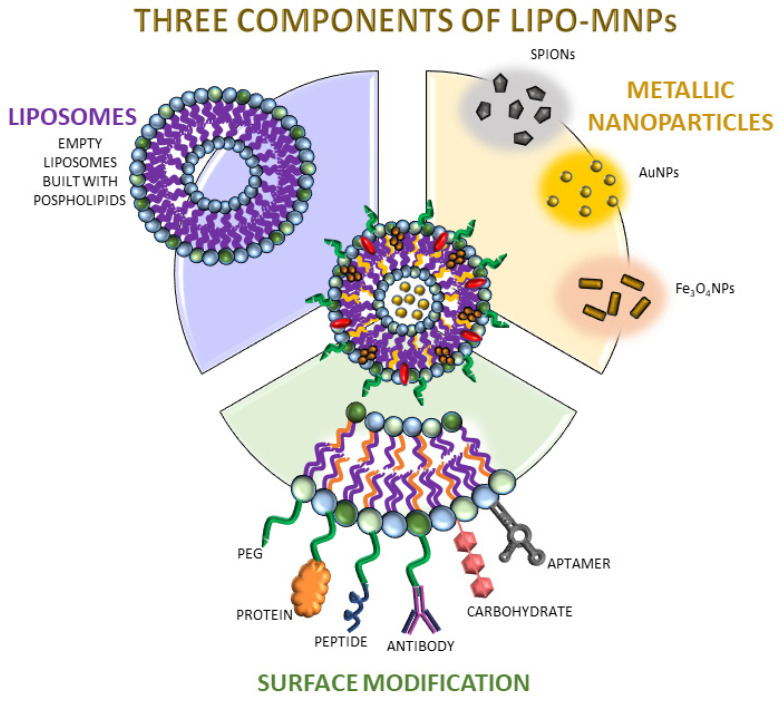
The scheme of liposomal modifications used in therapy support or medical imaging. Three of the most important components were highlighted as crucial parts of LipoMNPs. It presents the empty liposome, which is built with phospholipids, the core of nanostructures containing metallic nanoparticles, and different kinds of surface modifications. Each of these components play a key role in LipoMNPs and has various impacts depending on its target usage. The Lipo-MNPs consist of phospholipids, cholesterol, DSPE-PEG, and MNPs.

Liposomes can be synthesized by different methods like the hydration film method [13], and then modified by incorporating various substances, e.g., metallic nanoparticles [13,14,15,16], or functionalized by different ligands, e.g., aptamers [17] (Figure 2). The combination of liposomes and metallic nanoparticles proved to be a potential technique that supports efficient medical imaging and treatment [18]. They have become the object of interest in the field of medical imaging, in particular for transporting contrast agents (CAs). There is a constant effort to improve the accumulation of contrast in the required area for better visualization. Research in this area has achieved a sophisticated level that is carefully suited to the diagnostic and treatment simultaneously. Particular attention is paid to the role of liposomes as adjuvants to the already known conventional therapies [19]. When designing liposomes, attention should be taken to avoid non-specific interactions, represent specific target binding, maintain stability and circulation time in the bloodstream as long as possible, escape the reticuloendothelial clearance system (RES), and avoid mononuclear phagocyte system (MPS) or penetration into the tumor interstitial fluid of TME with high pressure [20].

Appropriate functions and their properties should be considered depending on the liposomes’ target application. Certain drug delivery systems are commonly used in the clinic. One example is Doxil^®^, a polyethylene glycol (PEG) liposome containing Doxorubicin the first nano-drug approved by U.S. Food and Drug Administration (FDA) [21,22,23]. It has been approved for the treatment of Kaposi’s sarcoma and ovarian cancer [24]. Its admission as the first nano-drug opened the way to the use of other such systems [25,26,27,28].

Conversely, structures designed for a drug delivery system will behave differently compared to those intended for imaging. Unfortunately, the combination of such nanostructures as liposomes and metallic nanoparticles is not widely used due to numerous limitations and the poorly understood nature of such compounds. There is a knowledge gap regarding the most efficient systems that could simultaneously image and treat cancer-related areas. There is no taxonomy that would allow the results to be compared in order to be able to design more efficient and improved systems.

Numerous reviews dedicated to liposomes have focused on a wide variety of biomedical applications [29]. However, the aim of this work is to present the most recent projects and published works focused on the use of advanced nanostructures such as liposomes in combination with metallic nanoparticles and other active substances such as drugs or contrast agents. We want to present their application in two areas: conventional therapy support and medical imaging. Especially, we want to focus on the advantages and disadvantages of this type of solution as well as indicate the further direction of work that should be undertaken to introduce such advanced nanostructures for clinical use.

## 2. Review Methodology

The literature search was conducted for papers related to metallic nanoparticles incorporated into liposomes as a new approach to the drug delivery system. Moreover, the search included papers dealing with using this kind of nanostructures in treatment and medical imaging. The publications were searched focusing on the PubMed database. The found articles were published in the years 1981–2021, which gives us a wide range of knowledge related to the presented topic of review. We used the following terms to find works cited in this review: “metallic nanoparticles in liposomes”, “gold nanoparticles incorporated into liposomes”, “metallic nanoparticles with liposomes in therapy”, “metallic Nanoparticles with liposomes in medical imaging”, “lipo-metallic nanoparticles”, “metallic nanoparticles in combined therapy”. We have established one main criterion for selecting a publication. The chosen articles should only be concerned with drug delivery systems consisting of metallic nanoparticles embedded in liposomes. The exclusion criterion was related to systems consisting of liposomes and attached drugs, or substances that were not metallic nanoparticles. Although there are many other complex nanostructures consisting of various components, our goal was to present only works focused on the medical therapy and imaging use of nanoparticles–liposome nanostructures.

## 3. Treatment

Here, we would like to focus on liposomes used in the transport of drugs and substances responsible for increasing the toxicity of conventional therapies. It is important to know all the crucial aspects, advantages, and limitations in order to properly design new cancer treatment strategies. One of the most frequently proposed methods involves the use of a combination of liposomes with metallic nanoparticles [30]. It is important to maintain appropriate conditions during synthesis and plan the effectiveness of such nanostructures, to obtain efficient transport to cells and the drug-releasing after reaching the target [31]. A challenge in this field of nanotechnology is the efficient incorporation of the drug into the liposome and the selection of the stimulating factor that does not destroy healthy tissues at the same time [32]. These types of advanced nanostructures have many biomedical applications. They are used for the controlled release of molecules or plasmids [33], cell activation [34], disease treatment [35], and imaging [36]. However, such strategies come with potential limitations. Very low drug incorporation is up to 5% [37]. The amount of drug administered is not effective enough to obtain a pharmacologically effective concentration in the body. Moreover, a significantly large amount of the drug may be released during transport before the liposome reaches its target site in the body, resulting in lower treatment activity and toxicity to healthy tissues [38].

**Figure 3:**
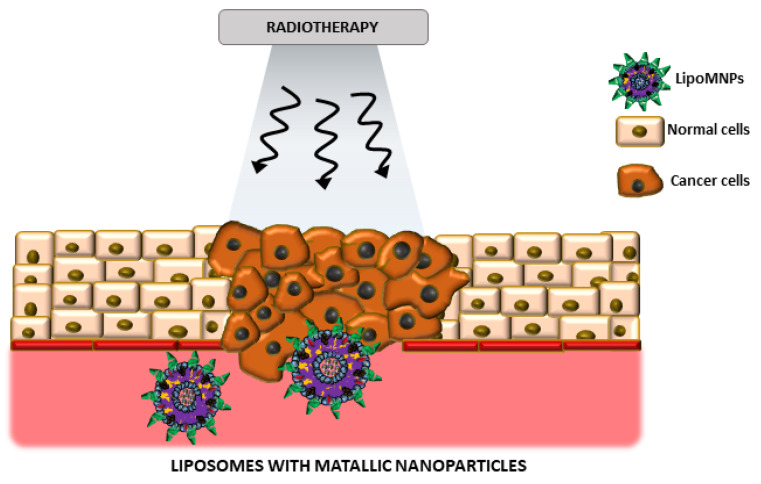
The schematic representation of LipoMNPs (Liposomes with metallic nanoparticles) usage as radiosensitizers in radiotherapy. Arrows are symbols of photons in ionizing radiation during radiotherapy.

The main interest of scientists is curing cancer due to the numerous difficulties that arise during the treatment process. Conventional treatment of cancer includes surgery, chemotherapy, and radiation therapy. Many different factors are used to sensitize cancer cells to ionizing radiation while protecting the percentage of normal cells in the field of the therapeutic beam [39]. Metallic nanoparticles are usually used as agents sensitizing cancer cells to ionizing radiation during radiotherapy [40]. In combination with liposomes as nanoparticle delivery carriers, it is possible to obtain a high-throughput treatment strategy. Success factors of this type of treatment system should also be taken into account [37]. Radiosensitizers incorporation must not have a destructive effect on the physical and chemical properties of the liposome. Usually, the process of realizing radiosensitizers is undertaken by using different factors. Under the influence of a stimulating factor such as temperature, pH or light energy, liposomes should decompose effectively. Moreover, the stimulus factor should not destroy healthy tissues (Figure 3).

The reason to look for improvements in conventional radiotherapy is the presence of hypoxia in the tumor area. Low oxygen concentration makes cancer cells significantly more resistant to the influence of drugs and ionizing radiation [41]. Moreover, hypoxia promotes the genetic transformation of cells into a more invasive phenotype, which further increases the predisposition to metastasis and disease recurrence [42]. In order to overcome those inconveniences, it has been proposed to use perfluorocarbons as O_2_ carriers [43]. These types of compounds are characterized by high incorporation and easier penetration abilities through abnormal vascular structures in the tumor microenvironment. Cheng et al. [44] presented encapsulated perfluorohexane within liposomes to form 100 nm nanoparticles. Their aim was to enhance the effect of photodynamic therapy by sensitizing the tumor cells in the bearing mice model. Researchers observed tumor growth inhibition by injecting a designed sensitizing agent. In vitro and in vivo studies, exhibited significantly higher effectiveness of Oxy-PDT compared to the conventional PDT method. The results from in vivo studies showed complete inhibition of tumor growth in mice using Oxy-PDT at low dose photosensitization together with 20 s laser irradiation. Conventional PDT did not achieve comparable results as the inhibition of tumor growth was minimal. The group presented very promising results that may constitute the potential efficacy of the undertaken cancer treatment strategy in photodynamic therapy.

In order to increase the biocompatibility and longer circulation time of liposomes in the bloodstream, they should be modified with poly(ethylene) glycol. Liu et al. [45] presented PEG-ylated liposomes to sensitize cells to radiation therapy. They proposed liposome-cholesterol-based nanoparticles conjugated with the radiosensitizer nitroimidazole by a hydrolyzable ester bond. Hybrid liposomes were introduced as a good carrier for promoting cargo release under hypoxic conditions. Doxorubicin-loaded liposomes were pharmacokinetically stable and scientists observed tumor growth inhibition using medical imaging. This experiment suggests that the hybrid liposomes are potential candidates as drug delivery systems for chemotherapeutics like doxorubicin, to improve the tumor response to treatment creating advanced synergistic chemo-radiotherapy. Zhang and coworkers [46] used cis-diamminedichloroplatinum(II) (cisplatin) attached to phospholipids as an oxygen generator in the tumor microenvironment. They highlighted the problems of implementation of the combined therapy in clinical usage. Catalase (CAT), an antioxidant enzyme, was incorporated in the liposome core constituted by cisplatin (IV)-prodrug-conjugated phospholipid, forming CAT@Pt(IV)-liposome. The designed structures caused a higher number of DNA double breaks after exposure of glioma cells to ionizing radiation. This approach integrated cisplatin-based chemotherapy and catalase-induced tumor hypoxia relief. Scientists have observed that their nanostructures are capable of accumulating sufficiently high in the tumor, while significantly reducing the hypoxia of hypoxic areas by triggering the decomposition of endogenous H_2_O_2_. It was found that CAT@Pt(IV) assessed the greatest results in combination with ionizing radiation. The designed “nano reactor” is a multifunctional structure with many benefits due to the simple method of synthesis and production, biocompatibility, good drug carrier, protecting the catalytic activity of the enzyme, and reduction of hypoxia. Their results constitute a good basis for further research in this direction, as the problem of resistance to radiotherapy and chemotherapy is still topical and difficult to overcome.

Gold nanoparticles (AuNPs) are the most frequently used to sensitize cancer cells. Authors [47] postulate, that radiosensitization with gold nanoparticles depends on the level of internalization. It takes place using two methods: micropinocytosis and passive diffusion membrane [48]. Chitchrani et al. [49] used liposomes as a “Trojan horse” to deliver 1.4 nm gold nanoparticles to cancer cells. Their aim was to overcome the energetically unfavorable endocytosis process for small nanoparticles. Researchers studied the efficiency of uptake and intracellular transport of fabricated nanostructures. The results demonstrated a 1000-fold enhancement of internalization using the liposome system. Moreover, the analysis showed that nanostructures made of liposomes and gold nanoparticles are delivered to the lysosomes after 40 min of incubation. This study provided much-needed information on the implantation of liposomes as a carrier of gold nanoparticles into the cell. Scientists [50] also investigated gold nanoparticle liposomes for their stability and the ability to release nanoparticles with light. Triggered drug release is a desirable form of substance administration due to its ability to control and regulate the release of the drug. The light energy is converted into thermal energy in gold nanoparticles, which is the factor causing the liposome breakdown. The aim of the research group was to analyze the drug delivery system and its release in the cytosol of the cell using visible light and near-infrared light signals. Moreover, scientists synthesized the liposome from components that were sensitive to changes in pH and temperature and incorporated gold nanoparticles in the form of rod and star shapes. Human retinal pigment epithelial cells (ARPE-19) and human umbilical vein endothelial cells (HUVECs) were used in this study. The designed nanostructures were non-toxic. The results demonstrated that the nanoparticles were not released without light interference. As a result, the light activated liposome formulations showed a controlled content release at the selected place and time.

Another research group [51] also used the combination of liposomes and gold nanoparticles, but to create a construction called the hybrid Cluster Bomb in the treatment of liver cancer. In addition, they used liposome-loaded paclitaxel in their study. The effectiveness of hybrid liposomes was proven by using xenograft Heps tumor-bearing mice. Researchers estimated the most efficient and effective ratio of individual components by presenting the accurate site and time-release mode for liver tumor treatment. They assessed the long-term effect of release on therapeutic efficacy by considering the results of a single dose for seven days by injection into the tail vein of mice. All paclitaxel (PTX) formulations were observed as effective in suppressing tumor growth after receiving a single dose. As assumed, the group treated with PTX/PTX-PEG400@GNPLips 1 (25:75) showed effective tumor inhibition. When comparing body weights between groups, it was found that the survival rate of the PTX/PTX-PEG400@GNPLips (25:75) treated group was significantly higher. At 7 days after injection, none of the mice died in the PTX/PTX-PEG400@GNPLips (25:75) group, while the survival rates in the saline and Taxol^®^ groups were only 50% and 70%, respectively. The results indicate that the combined strategy of drug burst and sustained release from the nano-delivery system enables the precise control of drug concentration in tumor tissue and cells to achieve better anti-tumor activity and reduced systemic toxicity. Sharifabad et al. [52] tested an equally advanced nanostructure, however, using superparamagnetic iron oxide nanoparticles. They created liposome-capped core-shell mesoporous silica-coated superparamagnetic iron oxide nanoparticles called “magnetic protocells”. Their aim was to prepare nanostructures and assess their toxicity on the MCF7 breast cancer cell line under the influence of an alternating magnetic field. Cells were treated with the synthesized compound and showed an approx. 20% decrease in proliferation compared to control cells. Researchers concluded that the use of their synthesis method would contribute to the treatment of tumors in animal models in the future in conjunction with magnetic hyperthermia. Bao et al. [53] designed the paclitaxel hybrid drug delivery system using liposomes and PTX-PEG400@GNPs gold nanoparticles. They used a covalent bond to conjugate thiol-terminated polyethylene glycol (PEG400)-PTX to gold nanoparticles and then incorporated the paclitaxel attached to the AuNPs into the liposomes. Due to this approach, the drug loading capacity increased. The encapsulated paclitaxel showed a longer circulation time and better targeting abilities than the commercial Taxol^®^ (BRISTOL MYERS SQUIBB, BMS, Warsaw, Poland). They observed also that the combination of organic and inorganic structures resulted in better effects than commercially used drugs. However, this method is associated with some drug release limitations as no statistically significant differences between the designed hybrids and the conventional drug were noticed in other research groups. This study provides a lot of information on how to improve synthesis methods and what factors should be considered designing new strategies. Xing et al. [54] also used liposomes and gold nanoparticles to deliver doxorubicin but in order to support photothermal synergetic antitumor therapy, nanoparticles and doxorubicin were locked inside the liposome. The authors observed that under the influence of NIR (Near Infrared) light, complex liposome/metallic hybrids are more absorbed by the endocytosis process because the structure of the liposomes was broken down and the gold nanoparticles easily reached the inside of the cell. The designed nanostructures showed an excellent anti-cancer effect, inhibiting the development of cancer cells by approx. 80%. Moreover, in the mice bearing tumor model, the nanostructures also presented themselves as tumor suppressors. A research group led by Zheng et al. [55] focused on the study of tumor-specific, pH-responsive, peptide-modified, liposome-containing paclitaxel and superparamagnetic iron nanoparticles PTX/SPIO-SSL-H_7_K(R_2_)_2_. Anticancer, imaging and targeting effect were assessed on the basis of an in vitro and in vivo model using the epithelial human breast cancer cell line MDA-MB-231. Their results confirmed the effectiveness of the produced nanostructures in selected issues, which constitute an advanced theragnostic approach in this field. Scientific publications about using LipoMNPs were collected in the Table 1.

The combination of metallic nanoparticles and liposomes has also been used in vaccine design. Scientists [58] explored a biomaterial-based approach of converting tumor-derived antigenic microparticles (T-MPs) into a cancer vaccine presenting their potential in multiple murine tumor models. They used adjuvant CpG-loaded liposomes with Fe_3_O_4_/T-MP on the surface to get a vaccine (Fe_3_O_4_/T-MPs-CpG/Lipo). The group demonstrated that the engineered, complex vaccine targeted to antigen-presenting cells induced a strong tumor antigen-specific host immune response. Moreover, the vaccine in the tumor microenvironment could alter tumor-associated macrophages into a tumor-suppressive M1 phenotype by nano Fe_3_O_4_, resulting in the transformation of a “cold” tumor into a “hot” tumor. This study exemplifies the modulation of the tumor immunosuppressive network which potentially constitutes a personalized vaccine cellular strategy.

The future clinical success of nanoparticles depends on a more detailed understanding of the mechanisms of their physicochemical properties on the biological response. Researchers have investigated many studies to obtain nanostructures resulting in efficient cancer treatment, but further research is desired [56,57,59]. The nanoparticle-based radiosensitization method is used in proton and particle therapies [60,61]. However, these methods are based mainly on the wide use of metal nanoparticles, therefore the possible use of liposome-metal nanoparticle systems in the future is fully open. In our opinion, liposomes containing gold nanoparticles represent a potential factor that is a good complement to conventional therapies. Such nanostructures can be used as drug carriers in chemotherapy and at the same time make cancer cells more sensitive to radiation therapy. Using Lipo-MNPs, a new therapeutic approach in the form of radio-chemotherapy can be designed.

## 4. Medical Imaging

**Figure 4:**
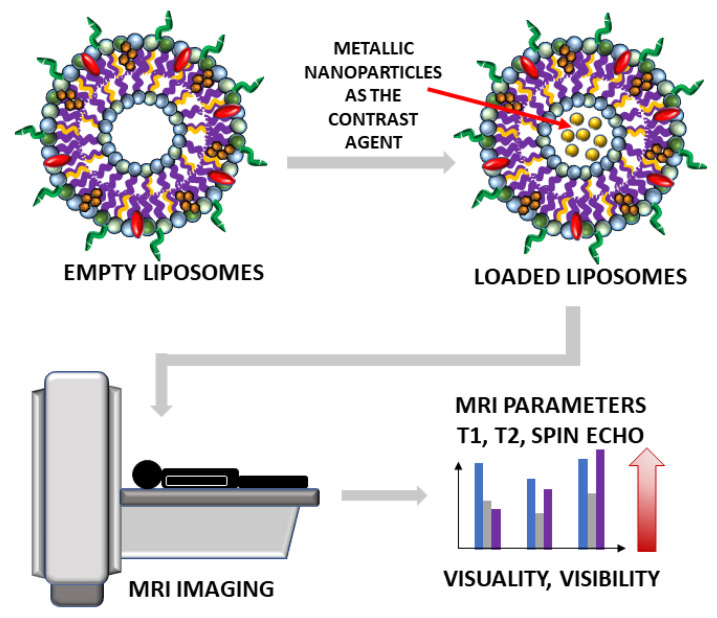
The scheme of future usage of liposomes loaded with metallic nanoparticles as the state-of-the-art contrast agents in medical imaging of patients will enhance the parameters of visuality and visibility. The red arrow is a symbol of increasing visuality and visibility in MRI imaging after nanostructures implementation.

Lipid nanoparticles have proven to be excellent potential transporters of agents for imaging and therapy [13,62,63]. In order to create the best liposomal structure, it is mandatory to understand and know their behavior in vivo. Therefore, there is a constant effort to improve visualization, quantification, and monitoring of liposomes biodistribution using non-invasive imaging techniques that could further give information about drug release, for example. To image nanoliposomal structures in vivo, labeling ions are incorporated into the liposomal membrane, bound to the membrane, or are enclosed in the liposome interior. There are several contrast agents (CAs) widely used in radiological and nuclear medicine preclinical and clinical routine, improving the image quality of non-invasive imaging techniques [64]. In the group of radiological CAs, one can mention gadolinium-based, manganese-based, iron oxide, and iron platinum contrast agents for magnetic resonance imaging (MRI) [65], iodine- and lanthanide-based as well as gold, bismuth, and other metals used as X-ray-computed tomography (CT) contrast agent [66] and phospholipids with sulfur hexafluoride, octafluoropropane, or perfluorobutane for ultrasonography (USG) [67] (Figure 4).

Nuclear medicine (NM) uses radionuclides for the labeling of specific ligands (radiopharmaceutical) that are trapped and metabolized by cancer cells. That makes possible both functional imaging and therapy if certain conditions are met. Depends on the imaging technique, radionuclides decay with beta plus process like gallium-68 (^68^Ga) or fluorine-18 (positron emission tomography, PET), or emit gamma rays at defined energy levels via isomeric transition or electron capture process like technetium-99m (^99m^Tc) or iodine-123 (^123^I) (single-photon emission computed tomography, SPECT; planar scintigraphy) emitting two or one gamma photon, respectively. The number of radiopharmaceuticals used for imaging is seemingly large [68]. However, going deeper into the subject one can recognize that this group is not so tremendous. The combination of liposomes and metallic nanoparticles used as contrast agents for mentioned technique proved to have the potential to supports efficient medical imaging. Research in this area [31,32] has achieved a sophisticated level that is carefully suited to the diagnosis and treatment. Although, most of the radiological modalities are freely available and used CAs are much cheaper than the production of radionuclides and their use for PET and SPECT scanning, their combination with liposomes suffers mostly from low sensitivity and signal to background. Contrary, nuclear techniques benefit from whole body capabilities, quantification, high sensitivity, and absence of tissue penetration issues, but presents limited spatial resolution [68]. For nuclear techniques, radiolabeling of liposomes was widely described in the literature [13,69,70,71] with the use of several different labeling methods [72]. According to Man et al. [68], in 2019, Tc-99m was the most commonly used radionuclide for liposome labeling. The second was indium-111 (In-111) and then slowly PET radionuclides started to play an important role with—copper-64 (Cu-64), manganese-52 (Mn-52), and zircon-89 (Zr-89).

The main approach which presented advantages of using LipoMNPs was introduced by German et al. [73]. The group assessed the efficiency of hybrid nanosystems (magnetite nanoparticles and liposomes, MFLs) as contrast agents. The magnetite nanoparticles (MNPs) were synthesized using the chemical precipitation form of Fe (II) and Fe(III) salts solution in a basic environment [74]. They showed their ability to perform MRI contrast enhancement in vitro and in vivo using renal carcinoma cells transplanted into rats during the administration of MFLs into the tumor. The scientists subcutaneously administered the suspension of renal cell carcinoma (Blokhin Russian Cancer Research Center) cells into twenty male albino Wistar rats. After the MRI study tissue samples of internal organs of the rats were taken for histological examination to assess the toxicity of the new contrast agent, the results revealed that MFLs increase the T1 (transverse relaxation time) parameter and decrease the T2 (longitudinal relaxation time). According to the authors, MFLs are able to effectively increase the contrast on T2-weighted images, allowing visualization of the tumor under both T1 and T2 sequences. The MFLs did not induce any significant changes in the internal organs due to MFLs’ biocompatibility.

The newest reports [75,76] suggest using liposomes in the area of imaging diagnostics and treatment in neurology. However, it is worth mentioning that the interest in this topic started much earlier when Vieira et al. presented a wide review summarizing accomplishments in the field of liposome-based drug delivery across the blood–brain barrier [77]. Authors have concentrated on liposomal strategies for imaging drug accumulation for the treatment of ischemic zone and glioma diagnosis. Moreover, the advantage of the presence of targeting ligands over the liposomal surface was emphasized as an improvement of the agent retention time in the tumor and its uptake by the cancer cells. Recently, various neurological studies focus on Alzheimer’s disease and amyloid plaque visualization [78], lesions in the brain due to HIV infection [79], Parkinson’s disease [80], and stroke [76].

Comparing various medical topics using liposomal delivery systems for imaging and therapy, oncology is the area with the biggest collection of data referring to its broad application. Liposome-nanoparticle hybrids that include metallic components such as: gold nanoparticles [75,81] or gadolinium nanoparticles (GdNPs) [63,82,83], and ultrasuper- and superparamagnetic iron oxide nanoparticles (SPIONs)–called magnetoliposomes (MLs) [60,84,85]—can serve in the visualization of the cancer tissues using CT and MRI. Liposome-based CAs incorporating gadolinium were the first introduced nanostructure that has been widely used in various systems [86,87,88]. Scientists constantly broaden their research in terms of the administration of newly synthesized contrast agents containing liposomes and metallic nanoparticles into different types of cancer tissues.

Breast cancer was chosen by several authors as the target for possible imaging with the use of liposome-nanoparticle hybrids. Experts agree that mammography is the best available diagnostic tool for the early detection of breast cancer in patients of risk. Mammography is followed then by biopsy and MRI increasing costs of diagnosis. For that reason, scientists started to look for other alternatives to mammography showing better sensitivity and specificity [89]. One of them was computed tomography [90].

One year later, the same author [91] published an in vivo analysis of biodistribution and pharmacokinetics performed using Swiss albino mice proving at the same time multifunctional capabilities of Lipos Au NPs and their hepatic-biliary and renal clearance. Lipos were synthesized following the identical protocol [19] with small modifications to achieve a smaller size of liposomes. The estimated mean diameter of the whole structure was 100–120 nm, where the coating was created by gold nanoparticles of the 5–8 nm diameter ensuring the renal clearance after biodegradation of core liposome [92]. Lipos Au NPs were injected i.v. ∼110 μg/400 μL through the tail vein and the analysis of various tissues, plasma, and urine was performed on days 1, 7, and 14 after injection. The authors observed that the injected particles were accumulated at the highest rate in the liver and spleen. A smaller percentage of 2–8 nm particles was accumulated in the kidney and further removed with urine. An in vivo study was carried out using the HT1080-f luc2-turboFP tumor xenograft model in BALB/c NUDE mice. When the tumor was around 70 mm^3^ large, mice were segregated into three groups of five animals and treated as followed: the first group by injection of normal saline, the second was treated with laser only, and the third with laser and by injected Lipos Au NPs (0.5 μg/μL in 30 μL). Results of the experiment showed that the bioluminescence signal was significantly lower on day 30 in group II and III compared to group I. Additionally, in four out of five animals in group III, tumor regressed completely comparing to animals from group I or II which all died naturally.

The interest of breast cancer diagnostics was also focused on MRI and its CAs, namely superparamagnetic iron-oxide nanoparticles creating negative contrast in T2-weighted images (spin-spin relaxation time) and gadolinium providing positive contrast in T1-weighted images (spin-lattice relaxation time). SPIONs have shown great potential in theragnostics applications like therapy (hyperthermia cancer treatment), targeted drug delivery, and separation of biomolecules. They respond nicely to external magnetic fields that allow for directing them specifically to the region of interest. It was shown that SPIONs smaller in diameter than 10 nm, act as T1 agents whereas bigger than this acts as T2 [85]. Therefore, SPIONs size control is a critical challenge for their application in MR imaging. Regarding the agglomeration problem, it can be controlled by coating SPIONs, e.g., with polymers or lipids [92].

He et al. [70] conducted an experiment using magnetic iron-oxide nanoparticles (MIONs) bound to liposomes including chemotherapeutic anticancer drug mitoxantrone (Mit) creating gonadorelin-functionalized Mit-loaded MLs (Mit-GML). They synthesized such hybrids in order to assess their effect in both therapy and MRI imaging of human MCF-7 breast cancer cells implanted into living animal models. A lipid film hydration method was used to prepare MION-loaded liposomes (MLs). They used the female athymic nude BALB/c mice with MCF-7 breast cancer cells implanted into their mammary fat pads. Mice underwent MRI examination after the tumor diameter reached 7–10 mm, with an aim of demonstrating the diagnostic ability of Mit-GML for targeted detection of cancer lesions. Images were obtained before and 2 h after contrast injection by a clinical MR scanner (GE Signa excite 1.5 T). Mit-GML significantly induced the targeted delivery of Mit to tumor cells with overexpression of LHRH receptors which resulted in inhibition of tumor cell growth. Results demonstrated an enhanced tumor accumulation of Mit-GML compared to other cells while analyzing biodistribution of the liposomes in various organs over 24 h. In a concentration-dependent manner in vitro the T2-weighted MR image of Mit- GML presented a dark MR contrast signal. The in vivo MRI T2-weighted images performed 2 h post-injection showed a decrease of signal intensity in the tumors. Cancer theranostics with Mit-GML presents a new strategy in breast cancer treatment with a strong need for further investigation.

Zhang et al. [93] created another combination of liposomes and metallic nanoparticles in the imaging diagnosis of breast cancer cells. They synthesized polyethylene glycol-coated liposomes. After that, SPIONs were incorporated into the core of liposomes and near-infrared dye (DiR) into the lipophilic bilayer creating hybrid nanostructures (PGN-L-IO/DiR). The authors used PGN635, a human monoclonal antibody that specifically targets phosphatidylserine (PS). They conjugated the antibody to the distant terminus of the polyethylene glycol chain. In vitro targeting specificity and toxicity was characterized with the use of adult bovine aortic endothelial cells (ABAE). Breast cancer cells MDA-MB-231 were injected subcutaneously on a thigh or both thighs of anesthetized mice. In vivo T2-weighted FSEMS MRI images were acquired at 9.4 T, acquired before and at different time points after i.v. injection of PGN-L-IO/DiR. After MRI at 24 h, near-infrared fluorescence imaging was performed and repeated at 48 h. The results confirmed the potential of the targeted liposomal–SPION hybrid for sensitive in vivo imaging of breast cancer cells in mice in a direct and accurate manner. The tumor visualization after i.v. infusion of PGN-L-IO/DiR was confirmed by both MRI examination and optical imaging. Irradiation resulted in an increase of PS exposure in MDA-MB-231 cells which confirms the use of this technique to facilitate the targeting of PGN-L-IO/DiR. The results provide perspectives for implementing both imaging and therapeutic agents into the PS- targeted liposomal platform. Recently, Patil-Sen et al. [94] reported on the fabrication of a three-component composite-magnetoliposome consisting of a SPION-silica core-shell coated with mesoporous silica and/or outer lipid layer. The aim of the construction of those structures was to show their potential theragnostic applications in drug delivery and magnetic hyperthermia. The capability of particles for hyperthermal therapy was also confirmed. Lipid coating enhanced the dispersion stability and lipid silica-coated SPIONs displayed significantly shorter T2 time as compared to the bare-SPIONs. The doxorubicin (DOX) release studies showed an efficiency of 35% for lipid-coated and 58% for silica-lipid-coated SPIONS. For the in vitro cytotoxicity study, the human fetal glial had a normal cell line (SVG) p12 and human breast cancer had a commercial cell line (MCF-7). Lipid-coated nanoparticles (without DOX) have shown excellent biocompatibility in vitro, against both cell lines. DOX-loaded nanocomposites showed high efficiency and selectivity as drug carriers against MCF-7 cell lines as compared to SVG p12 (indicative result). All those results show that a combination of lipid–silica dual coating for SPIONs creates a potential theragnostics system that can be used for both, diagnostic and targeted drug delivery, in hyperthermia-mediated cancer therapy.

A recent example of metallic liposomes use for breast cancer studies in nuclear medicine can be Lee et al. [95]. They reported a clinical trial of HER2-targeted PEGylated liposomal doxorubicin, labeled with Cu-64 radioisotope (Cu-64-MM-302) showing the enhanced permeability and retention effect in relation to treatment response of HER2-positive metastatic breast cancer. The research was driven in a cohort of 19 patients who had administrated Cu-64-MM-302 structures and afterward were imaged using PET/CT. Results showed that particles circulated for over 24 h and accumulated mostly in the liver and spleen. Retrospective analysis of outcomes related to the delivery of the drug into the tumor lesions and its high deposition were associated with more favorable treatment outcomes (HR = 0.42).

The imaging diagnostics of other cancers common in women that drew the attention of the scientists was visualization and monitoring of the therapy effects on ovarian cancer. According to Bray et al. [96], ovarian cancer is one of the most common cancers risking women’s health and has the worst prognosis and mortality rate. Ravoori et al. [97] investigated a new contrast agent that could perform in both staging in the pre-surgery planning under MRI and optical aid in the surgical operation. They synthesized dual gadolinium liposomal contrast agent (DM-Dual-Gd-ICG) and used human ovarian cancer HeyA8 cells and OVCAR-3 cells injected into nude female mice. Increased T1-weighted MR signal and NIR signal were visualized in mice tumors injected with DM-Dual-Gd-ICG after two days. Results suggest potential clinical use in multimodal imaging that can be beneficial in the diagnosis and treatment of advanced ovarian cancer stages.

Chen et al. [98] synthesized a novel molecular imaging nanoprobe with the ability to perform as a dual modality in MRI and NIRF. The most important integrin for angiogenesis in many solid tumors, e.g., liver cancer, indocyanine green (ICG), was used as a NIRF dye. The synthesized probe consisted of SPIO@ Liposome bound to ICG and RGD (peptide: arg-gly-asp). They used HepG2 liver cancer cell lines for cytotoxicity assessment as well as integrin binding. In vivo imaging was performed in Balb/c male nude mice with a subcutaneous tumor acquired from injecting HepG2 cells. Scientists distributed intravenously the SPIO@Liposome-ICG-RGD in mice and observed their effect in both fluorescence imaging and MRI with a dual-modality technique. The study showed clear tumor delineation after SPIO@Liposome-ICG-RGD probe injection. The contrast-to-noise ratio obtained from MRI was helpful for detecting smaller tumors (0.9 ± 0.5 mm). The small tumors appeared to be much brighter than the surrounding normal liver tissue while using the fluorescence surgical navigating system. SPIOs represent the first nanoparticle MRI contrast agents in clinical use and show excellent biocompatibility and performance in the visualization of the tissues. Moreover, ICG is the only NIR dye approved by the FDA for diagnosis in clinical applications. The authors suggest a profound value in clinical practice by stating an example of patients with liver cancer exhibiting similar degrees of metastasis.

Very interesting results of gadolinium–lipid complexes containing PE-DTPA (chelating Gd+3) are presented by Šimečková et al. [99]. Structures were prepared using the lipid film hydration technique. Their cytotoxicity was tested in human liver cancer HepG2 cells, liver non-differentiated progenitor HepaRG cells, and HepaRG cells differentiated into both, hepatocyte-like and biliary epithelial cell populations. No toxicity and side effects were detected indicating the potential safety of the synthesized liposomes. Lorente et al. [100] chose colon cancer as a target of theragnostic therapy. Their MLPs (γ-Mag-NP-LPs) were synthesized using maghemite nanoparticles (γ-Fe2O3) incorporated in phosphatidylcholine (PC) liposomes. These magneto liposomes were tested in the human colon fibroblast CCD-18 cell line, human colon carcinoma T-84 cell line, and the murine macrophages RAW cell line. The cytotoxicity of NPs in macrophages, lymphocytes, and erythrocytes was also tested. The authors observed γ-Mag-NP-LPs internalization into tumor cells with the use of transmission electron microscopy (TEM) and Prussian Blue staining. In order to determine qualitatively the magnetic-induced mobility of cells treated with *γ*-Mag-NP-LPs, authors used T-84 cells. After 24 h, cells were treated with 10 and 100 μg/mL of *γ*-Mag-NP-LPs. The results presented satisfactory magnetic and biological properties of the MLPs in vitro, including a high degree of biocompatibility. Biosecurity in clinical use was confirmed by analyzing the ability to produce lysis of human blood cells and no increase in lysis was observed. The results suggest that the properties of γ-Mag-NP-LPs, make them acceptable for their loading with chemotherapeutic drugs or for hyperthermia treatment. In 2017, Blocker [101] published results of MM-DX-929 (Merrimack Pharmaceuticals, Inc., Cambridge, MA, USA) liposomes labeled with Cu-64 (64Cu-MM-DX-929) for PET imaging. The aim of the study was to capture the image of the effect of short-term Bevacizumab treatment on liposome delivery to the colon tumor. In vivo studies were carried out in HT-29 human colorectal adenocarcinoma tumors grown in severe combined immunodeficient mice (SCID). Animals received around 200–300 μCi of 64Cu-MM-DX-929 (20 μmol/kg lipid) i.v. via the tail vein. PET imaging was followed by CT scanning for the same period of time. As a consequence, authors showed that positron emission tomography can detect significant differences in liposome delivery to treated colon tumors when compared to untreated controls using MM-DX-929 labeled with Cu-64.

Thebault et al. [102,103] presented a novel approach with the implementation of theragnostic MRI liposomes for cancer imaging. In the work [102], the authors aimed to evaluate the magnetic targeting (MT) efficiency of synthesized ultra-magnetic liposomes (UML) for MRI imaging. They used CT26 murine colon tumor in Balb/C female mice. The MT method allowed an efficient semi-quantitative evaluation of targeting efficiency in tumors with a possibility of implementing this technique to different T2 contrast agents. In their next study [103], authors proposed a very compelling therapeutic strategy that consisted of the magnetic accumulation of thermosensitive ultra-magnetic liposomes encapsulating iron oxide nanoparticles (UML) and an antivascular disrupting agent combretastatin A4 phosphate (CA4P). They used the High-Intensity Focused Ultrasound (HIFU) technique to trigger the release of CA4P in a colon tumor. The whole process was monitored by MRI examination. Firstly, scientists studied the effect of CA4P-UML and their ability to damage the cytoskeleton of EAhy-926 endothelial cells. In order to evaluate the perfusion in the tumor 24 h after the treatment, the authors performed a Dynamic Contrast-Enhanced DCE perfusion protocol. CA4P-UML showed a dual effect of magnetic targeting and MRI monitoring. The full combination consisting of UML containing CA4P with magnetic targeting and local ultrasound to trigger the CA4P release provided a statistically significant decrease of the tumor volume. MRI bioimaging proved the benefits of this therapy in vivo with the functional decrease of the vasculature in terms of permeability as well as the reduction of growth of the tumor. Scientific publications about using LipoMNPs in medical imaging were collected in the Table 2.

In our point of view, Lipo-MNPs are much more often used in medical imaging than in therapy. Such nanostructures can be observed in almost all commonly used imaging due to the metallic core of the nanostructures. This design allows for better results and imaging parameters. However, we have noticed that the conducted research is also associated with many limitations, which should be taken into account in the future.

## 5. Future Perspectives

The combination of liposomes and metallic nanoparticles has allowed the practical design of nanoscale devices combined with numerous functional molecules, including tumor-specific ligands, antibodies, anti-cancer drugs, and imaging probes. Moosavian et al. [30] presented the challenges and limitations in the development of liposomal drug delivery systems in anti-cancer therapies. They emphasized that researchers, in the future, should focus on better preparation of liposomes that would be more efficient in clinical trials. Moreover, it was suggested that results obtained on selected animal models are overestimated and show inadequate effects of liposomes, which will not occur in clinical implementation. A similar tendency can be observed in the above-presented works related to the use of systems consisting of liposomes and metallic nanoparticles. None of the teams considered several models or the optimization process of the method of obtaining liposomes. It would be recommended to describe the differences between individual variants of liposomes that differ in size, composition, or other biofunctionalizations. Moreover, researchers focus only on describing the effect of therapy of liposomes on cancer cells without taking into account the description of their biointeractions with biological matter [105]. Considering the radiosensitization effect of metallic nanoparticles on cells, it is important to clear the spatial relationship between the distribution of oxygen molecules and hypoxic tumor regions. It is necessary to optimize the conditions and time of reoxygenation in the area of the tumor microenvironment after the application of active nanostructures and radiotherapy. The relationship between liposome internalization and hypoxia is still unknown.

Furthermore, scientists should focus on targeting the transport of nanostructures towards specific cell organelles. Most studies only consider liposome targeting due to ligands and receptors on the surface of cancer cells. This is a logical approach to target engineered nanostructures directly to enter the cell, but liposome performance inside the cell is poorly understood. Hybrid liposomes action should show selectivity for given organelles depending on the desired effect. Researchers observe that nanoparticles released from the liposomes should reach the mitochondria to impair the functioning of the cell, or directly the nucleus, especially in gene therapy or in the regulation of protein metabolism.

We assume that in the future, scientists should use more models in experiments, using different cell lines, normal and cancer ones. However, when doing in vivo experiments, they should also focus on the influence of nanostructures on healthy tissues, highlighting the given key parameters that have a selective effect between normal and cancer cells. In addition, more attention should be paid to the more careful planning of fabricated nanostructures in order to be able to present the reasons why one component was used for synthesis. Our conclusions should help scientists learn from previous studies and plan better, more refined treatment or imaging strategies.

## 6. The Various Development Methods of Lipo-MNPs

The nanotechnology era has introduced various novel synthetic strategies in the area of controlled drug delivery. Liposomes are attractive biomimetic nanocarriers characterized by their biocompatibility and high loading capacity [106]. The constantly growing literature database in the field of liposomology, covering complementary studies from different fields, is an indication of increasing interest in this research area. Many techniques and methodologies have evolved for the preparation of liposomes, on small and large scales, since their introduction to the scientific community around 40 years ago [107]. Incorporating drug molecules into nanocarriers offers exciting opportunities to redefine the pharmacokinetic behavior of the drug, improving its therapeutic efficiency and reducing side effects [108,109,110]. Several types of drug delivery nanocarriers based on organic platforms such as liposomes, polymers, and dendrimers have been used as “smart” systems that can release therapeutic agents under physiological conditions [109]. Synthetic liposomes are small artificial vesicles of spherical shape that can be created from cholesterol and natural nontoxic phospholipids. Liposome properties differ considerably with lipid composition, surface charge, size, and the method of preparation [M1]. These nanostructures increased the efficacy and therapeutic index of the drug, stability via encapsulation. They are non-toxic, flexible, biocompatible, completely biodegradable, and non-immunogenic for systemic and non-systemic administrations [111].

We have highlighted here various methods of liposome preparation. General methods of preparation include four basic stages: (1) drying down lipids dissolved before in the organic solvent, (2) dispersing the lipid in aqueous media, (3) purifying the resultant liposome, (4) analyzing the final product (polydispersity, stability, etc.) [112]. It should be noted that only a few of the conventional liposome preparation procedures are capable of entrapping large quantities of water-soluble agents [113]. Bioactive agents can be entrapped in lipid vesicles by the conventional methods of reverse-phase evaporation technique [114], ether injection/vaporization technique [115,116], and freeze-thaw method [117], just to name a few.

While there are already existing drug delivery systems—e.g., liposomes, available for treatment—the effective loading and retention of the expected drug ratio can be challenging. Liposome preparation protocols vary depending on their intended application. Moreover, the continuous development of synthesis methods and the advancement of the resulting nanostructures is noticeable. In addition to conventional liposome synthesis methods, we would like to present examples of other systems consisting of metals and liposomes. Illes et al. [118] proposed a new type of drug carrier: liposome-coated metal-organic framework (MOF) nanoparticles. These systems combine the advantages of liposomes with an easy and efficient loading process. They also presented the successful synthesis of liposome-coated MOF nanoparticles via the fusion method. The resulting nanostructure, once loaded, show no premature leakage and an efficient release. The successful loading of these nanovehicles with both single and multiple drugs at the same time makes them a promising candidate for use in combination therapy. Wuttke et al. [119] presented novel metal–organic framework nanoparticles encapsulated by a lipid membrane. They demonstrated that the MOF@lipid system can effectively keep dye molecules inside the porous scaffold of the MOF while the lipid bilayer precludes their premature release. The group employed fluorescence microscopy in order to demonstrate the high uptake of lipid-coated nanoparticles by cancer cells. Considering the various ways to synthesize different functionalized MOF nanoparticles as well as the richness of lipids with diverse functions, MOF@lipid nanoparticles have great potential as a novel hybrid nanocarrier system. It is worth noting that, the MOF core is capable of storing different active ligands such as drugs or molecules used for imaging and diagnosis. Moreover, the lipid shell could be used for the incorporation of targeting or shielding molecules (e.g., PEG) as well as for the creation of controlled release mechanisms.

Metal nanocarriers are emerging structures that can enhance the therapeutic activity of many drugs by improved release and targeted potential, however numerous barriers, such as colloidal instability, cellular toxicity, and poor cellular uptake, restrain their applicability in vivo. The nanohybrid systems offer to overcome these limitations and to combine the properties of liposomes and metal nanocarriers for effective theragnostic drug delivery systems [106].

## 7. Conclusions

The term “theragnostic”, introduced in 1998 by J. Funkhouser, means the ability to combine therapy and diagnostics of disease at the same time. Determining this was an important step forward towards personalized medicine. The possibility of combining nanoparticles bound to liposomes with drugs offers a chance of implementing this idea of the approach to the disease [71,120]. LipoMNPs are a promising step forward in a more accurate visualization of the tissues and structures in imaging diagnostics [31]. It is possible because of the biological properties of liposomes and the ability of metallic nanoparticles to facilitate the imaging process [5]. Researchers in recent years analyzed such combination and their effect on several cancer cells of different types both in vitro and in vivo assessing the cytotoxicity and safety of this novel type of contrast agents [70]. The results [6] show the true potential of LipoMNPs in the novel approach to the idea of theragnostic treatment. Before an implementation to the clinic, there is a strong need for further investigation. Researchers should focus on multiple implementations of once synthesized liposome-metallic particles contrast agents in different types of cancer cells. There is a need for finding an optimal synthesis technique, resulting in a more universal hybrid that can be applied to different cancerous tissues with the ability to target drugs suited to the cancer characteristics. Future progress requires possible clinical trials preceded by numerous in vivo studies as well as more research towards the use of clinical diagnostic imaging machines in visualizing the liposome-nanoparticle hybrids [62]. Moreover, there is still a lack of knowledge about biointeractions between nano hybrids and cancer cells. The level of conducted experiments is advanced because investigators have designed new strategies on the basis of previous results.

## Figures and Tables

**Table 1:** Selected applications of liposomes with metallic nanoparticles in cancer therapy.

Author	Year	Nanostructures	Synthesis Method	Nanostructures’ Size	Cell/Tissue	Kind of Therapy	Results
Bromma [56]	2019	AuNPs entrapped in lipid nanoparticles	rapid-mixing method	53 nm	Breast cancer cells, MDA-MB-231	Radiotherapy	The addition of LNPs into tumor cells produced a 27% enhancement in tumor cell death
Bao [53]	2014	PTX-conjugated GNPs (PTX–PEG400@GNPs) in liposomes	thin film hydration	3.41 nm gold core	A murine liver cancer model	The drug delivery system	Maintains the superiority of both vehicles and improves the performance of hybrid systems
Chitchrani [49]	2010	AuNPs in liposome-based system	thin film hydration	105 nm	Cervical cancer cells, HeLa	The assessment of cellular uptake and transport	Au NP–liposomes demonstrated that they reside in lysosomes
Liu [45]	2020	Au nanoparticles and perfluorohexane nanoparticles encapsulated in lipid shell	film hydration method coupled with a double emulsion method	108 nm	Human anaplastic thyroid cancer cells, C643	Low-intensity focused ultrasound diagnosis ablation	An optional therapeutic platform for treating patients with drug-resistant cancer
Wang [57]	2017	Loading resveratrol (Res) in chitosan (CTS) modified liposome and coated by gold nanoshells (GNS@CTS@Res-lips).	mediation of CTS	115 nm	Cervical cancer cells, HeLa	Photothermal therapy	The nanocarriers displayed a synergistic antitumor effect of chemo photothermal therapy compared with PTT or chemotherapy alone
Zhu [16]	2018	Carboxyl-modified Au@Ag core-shell nanoparticles (Au@Ag@MMTAA) contained in the liposomes (DSPE-PEG2000-NH2)	thin film hydration	215 nm	Breast cancer cells, SKBR3	The assessment of cellular uptake and transport	The nanohybrids entered cells mainly through clathrin-mediated endocytosis and tended to attach on the cell, the highest mortality in vitro after laser treatment, surface before arriving in acidic lysosomes
Rengan [36]	2014	The Lipos Au particles	thin film hydration	100–150 nm	Breast cancer cells, MDA-MB-231	The drug delivery system and photothermal therapy	The efficient deployment for drug delivery application using NIR laser irradiation, enhanced parameters of drug delivery, and optical imaging, the Lipos Au NPs exhibited their true multifunctional ability by emitting good signals in CT X-ray analysis
Zhang [51]	2016	Gold conjugate-based liposomes with hybrid cluster bomb structure	thin film dispersion method,	115–150 nm	Xenograft Heps tumor-bearing mice	The multi-order drug delivery system	The time-release mode for tumor treatment using antitumor drugs
Sharifabad [52]	2016	Liposome-capped core-shell mesoporous silica-coated superparamagnetic iron oxide nanoparticles called ‘magnetic protocells’	lipid hydration	53 nm	Breast cancer cells, MCF7 and likely glioblastoma cells, U87	The drug delivery system	Loaded nanoparticles under alternating magnetic field exhibited nearly 20% lower survival rate of cancer cells
Zheng [55]	2018	liposome-containing paclitaxel (PTX) and superparamagnetic iron oxide nanoparticles (SPIO NPs), PTX/SPIO-SSL-H_7_K(R_2_)_2_,	thin film hydration	3.41 nm gold core	human breast cancer cell line, MDA-MB-231	The drug delivery system	Antitumor effect and enhancement of MRI parameters

AuNPs—Au Nanoparticles; LNPs—Lipos nanoparticles; PTX—paclitaxel; GNPs—Gold Nanoparticles; PEG—Polyethylene Glycol; GNS—gold nanoshells; PTT—Photothermal therapy; DSPE—1,2-Distearoyl-sn-glycero-3-phosphorylethanolamine; MMTAA—2-mercapto-4-methyl-5- thiazoleacetic acid; CT—computed tomography; SPIONPs—superparamagnetic iron oxide nanoparticles.

**Table 2:** Selected applications of liposomes with metallic nanoparticles in medical imaging.

Author	Year	Nanostructures	Synthesis Method	Nanostructures’ Size	Cell/Tissue	Kind of Therapy	Results
German [72]	2015	Magnetite nanoparticles (MNPs) in magnetic fluid loaded liposomes (MFLs)	chemical precipitation form of Fe(II) and Fe(III) salts solution in basic environment; extrusion technique	NPs: 13 nm MFL size: 147 nm	Renal cell carcinoma administered subcutaneously into twenty male Wistar albino rats	MRI	Increase of T1, decrease of T2 time; visualization of the tumor under both T1 and T2 sequences
He [104]	2014	magnetic iron-oxide nanoparticles (MIONs); gonadorelin-functionalized Mit-loaded MLs (Mit-GML)	lipid film hydration	Mit-GML size-136 nm	MCF-7 breast cancer cells implanted into female athymic nude BALB/c mice	MRI	Enhanced tumor accumulation of Mit-GML; 2 h post injection decrease in T2 signal intensity in tumors
Zhang [92]	2014	super-paramegnetic iron-oxide nanoparticles (SPIONs), hybrid nanostructures (PGN-L-IO/DiR)	lipid film hydration	PGN-L-IO/DiR size: 111 nm	MDA-MB-231 breast cancer cells injected subcutaneously into nude BALB/c mice	MRI	tumor visualization: 24 h post injection hypointense intratumoral regions appeared
Patil-Sen [93]	2020	composite-magnetoliposome hybrid: SPION-silica	co-precipitation, thin film hydration, surfactant templating approach	hybrid size: 150 nm	MCF-7 breast cancer cells, fetal glial normal cell line SVG -12	MRI	proven use as a negative contrast in MRI imaging;
Lee [94]	2017	PEGylated liposomal doxorubicin, labeled with Cu-64 radioisotope (Cu-64-MM-302)	no information	no information	HER2-positive metastatic breast cancer cells—19 patients	PET/CT	Particle tumor accumulation and visualization in imaging techniques
Ravoori [96]	2016	dual gadolinium liposomal contrast agent (DM-Dual-Gd-ICG)	lipid film hydration	<150 nm	HeyA8 or OVCAR-3 ovarian cancer cells, intraperitone-al injection	MRI	Increased T1-weighted MR signal and NIR signal in tumors
Chen [97]	2017	SPIO@ Liposome bound to ICG and RGD	film method followed by extrusion	<150 nm	HepG2 liver cancer cells subcutaneously injected into ten Balb/c nude mice	MRI	clear tumor delineation after probe injection, contrast-to-noise ratio helpful for detecting smaller tumors
Blocker [100]	2017	MM-DX-929 liposomes labeled with Cu-64	empty MM-DX-929 liposomes were provided by Merrimack Pharmaceuticals	104 nm	HT-29 human colorectal adenocarcinoma cells grown in SCID mice	PET, CT	MM-DX-929 labeled with Cu-64 detect significant differences in liposomes delivery to treated colon tumors when compared to untreated controls.
Thebault [102]	2020	CA4P-loaded thermosensitive Ultra Magnetic Liposomes (CA4P-UML)	co-precipitation method, reverse-phase evaporation method	209 nm	CT-26 murine colon tumor in Balb/C female mice	MRI	decrease of the tumor volume and vasculature observed in MRI

Abbreviations: ICD—indocyanine green, RGD-Arginine-Glycine-Aspartic peptide, CA4P—combretastatin A4, PEG-polyethylene glycol, MRI—magnetic resonance imaging, PET—positron emission tomography, CT—computed tomography PGN—human antibody PGN635, L-liposome, IO—iron oxide, DiR—near-infrared dye, SPION- super-paramegnetic iron-oxide nanoparticle, MION—magnetic iron-oxide nanoparticle, UML—ultra magnetic liposomes, MM-DX-929—64Cu-liposomal doxorubicin PET Agent (Merrimack Pharmaceuticals, Inc. Cambridge, MA, USA), MM-DX-302 -HER2-targeted antibody–liposomal doxorubicin conjugate (Merrimack Pharmaceuticals, Inc. Cambridge, MA, USA), SCID—Severe combined immunodeficient mice.

## Data Availability

MDPI Research Data Policies.
